# Dietary Fructose and Sodium Consumed during Early Mid-Life Are Associated with Hypertensive End-Organ Damage by Late Mid-Life in the CARDIA Cohort

**DOI:** 10.3390/nu16070913

**Published:** 2024-03-22

**Authors:** Dragana Komnenov, Mohammad Al-Hadidi, Hamza Ali, Malik Al-Jamal, Kassim Salami, Samy Shelbaya, Kareem Tayeb, Daniel Domin, Rana Elhamzawy

**Affiliations:** 1 Physiology and Internal Medicine, Nephrology and Hypertension, Wayne State University School of Medicine, Detroit, MI 48201, USA; gn6591@wayne.edu (M.A.-H.); go9572@wayne.edu (H.A.); maljamal@wayne.edu (M.A.-J.); khsalami@wayne.edu (K.S.); go9835@wayne.edu (K.T.); gp1614@wayne.edu (R.E.); 2 John D. Dingell VA Medical Center, Detroit, MI 48201, USA

**Keywords:** dietary fructose, dietary sodium, blood pressure, hypertensive target organ damage

## Abstract

We aimed to investigate how dietary fructose and sodium impact blood pressure and risk of hypertensive target organ damage 10 years later. Data from *n* = 3116 individuals were obtained from the Coronary Artery Risk Development in Young Adults (CARDIA) study. Four groups were identified based on the four possible combinations of the lower and upper 50th percentile for sodium (in mg) and fructose (expressed as percent of total daily calories). Differences among groups were ascertained and logistic regression analyses were used to assess the risk of hypertensive target organ damage (diastolic dysfunction, coronary calcification and albuminuria). Individuals in the low-fructose + low-sodium group were found to have lower SBP compared to those in the low-fructose + high-sodium and high-fructose + high-sodium groups (*p* < 0.05). The highest risk for hypertensive target organ damage was found for albuminuria only in the high-fructose + high-sodium group (OR = 3.328, *p* = 0.006) while female sex was protective across all groups against coronary calcification. Our findings highlight that sodium alone may not be the culprit for hypertension and hypertensive target organ damage, but rather when combined with an increased intake of dietary fructose, especially in middle-aged individuals.

## 1. Introduction

Cardiovascular disease (CVD) is the leading cause of death in the United States [1]. According to the American Heart Association’s Life’s Essential 8, a diet rich in fruits and vegetables and limited in added sugar and sodium is one of the eight pillars of cardiovascular health management [2,3]. Added sugars in processed foods such as fruit juices, bread and packaged goods consist of sucrose, which is a disaccharide made up of glucose and fructose or high-fructose corn syrup (HFCS), which contains 50% more fructose than glucose [4,5]. Studies completed on animals showed that even moderate increases in dietary fructose and sodium in the short-term result in elevated blood pressure at least partially via increased sympathetic tone, as well as the development of diastolic dysfunction and increased aortic stiffness [6,7,8]. Other rodent models showed that an increased ingestion of fructose and salt at an early age (i.e., equivalent to adolescence in humans) increased blood pressure, vascular stiffness and decreased glomerular filtration rate (GFR) later in life even after the exposure to fructose and salt had been removed and later re-introduced to rats once they were older (i.e., equivalent to mid-life in humans) [9,10]. Several human cohort studies have highlighted that dietary fructose contributes to obesity, insulin resistance and dyslipidemia, all of which are expected to impair cardiometabolic health [11]. Moreover, we recently published that both BMI and dietary fructose intake in individuals in their mid-forties are independent predictors of increased CVD risk 15 years later [12].

The consensus on whether escalating dietary fructose consumption is directly linked to cardiometabolic disturbances (i.e., diabetes) has not been reached [13]. However, evidence exists that fructose-enriched diet causes cardiovascular disturbances at the level of cardiomyocyte metabolism [14] and that elevated myocardial levels of fructose are associated with diastolic dysfunction in rats [15] and diabetes in human patients [15,16]. Studies in humans are lacking in examining the impact of dietary fructose on kidney function (i.e., albuminuria and GFR). However, some evidence exists linking serum uric acid, which is a downstream product in the fructose metabolic pathway, with a near 2-fold increased risk of albuminuria and rapid GFR decline in type-1 diabetic patients and 1.5-fold increased risk of coronary artery calcification progression [17]. Whether this is true in the non-diabetic population remains unknown. Likewise, consistent engagement in exercise may be important in mitigating the risk of developing CVD and kidney disease. In individuals with pre-existing cardiovascular disease, a rise in regular physical activity is linked to the regression of atherosclerotic plaques and an increased vasodilatory response mediated by the production of nitric oxide [18]. A study involving 4011 adults revealed an inverse relationship between physical activity/exercise intensity and the rate of rapid kidney function decline [19]. Additionally, individuals in the early stages of renal failure tend to engage more in sedentary behaviors, such as prolonged sitting and watching television, compared to those with adequate kidney function [19]. It remains to be determined whether there is a certain exercise duration/intensity threshold for these benefits and whether such habits could overturn the effects of dietary fructose and/or sodium on end-organ damage. In other words, can we outrun a bad diet?

Here, we set out to determine the relationship between dietary fructose and sodium, in combination and alone, with blood pressure and end-organ damage in a middle-aged cohort of Black and White males and females who are a part of the CARDIA (Coronary Artery Risk Development in Young Adults) cohort. CARDIA is an ongoing cohort study that was initiated in 1985 at four centers across the United States. The original cohort enrolled 5114 subjects in their early adulthood (18–30 years of age) who have been followed for 35 years and the cohort is biracial by design. Our findings and those of others from the CARDIA cohort have contributed importantly to the body of knowledge regarding the link of some dietary aspects, such as dietary sodium and low-carbohydrate diets with the coronary artery calcium progression [20] and fructose with CVD development [12]. Additionally, we recently demonstrated that sodium intake, but not fructose, in male CARDIA participants was significantly associated with a worse vascular aging index and that potassium intake and aerobic exercise were protective against vascular aging [12]. In the present investigation, we hypothesized that individuals who were in the upper 50th percentile for dietary fructose and sodium consumption would have higher blood pressure and higher risk of hypertensive target organ damage. 

## 2. Materials and Methods

### 2.1. Study Sample

The study sample consisted of the participants from the CARDIA study who were drawn from the general population across four centers in the US: Birmingham (AL), Chicago (IL), Minneapolis (MN) and Oakland (CA), matched for demographic factors such as sex, age, race (Blacks and Whites) and socioeconomic factors such as education level. The impetus for initiation of the study was to determine lifestyle and behavioral drivers of cardiovascular disease starting in young adulthood and extending through mid-life and beyond. The characteristics and details of the study were published before [21]. So far, a total of 9 examination cycles have been completed since 1985, occurring at 0, 2, 5, 7, 10, 15, 20, 25 and 30 year intervals. An updated outcomes status has been obtained for cycle 10 (year 35 of follow-up); however, the rest of the follow-up measurements have not been released yet as study visits had to be postponed given that the year of the scheduled visits coincided with the year of the global pandemic (COVID-19). Here, we considered the records from *n* = 3116 participants who attended examination cycle 9 (follow-up year 30) at which echocardiography was completed since most of our outcome variables were obtained from indices obtained with echocardiography scans. Therefore, no missing data were observed for all of the outcome variables. The overall missing data rate for other variables was no more than 5% and they were not imputed. The IRB approval (IRB #063319MP2X) was obtained by the Institutional Review Board at Wayne State University and the Research Materials Distribution Agreement by the National Institutes of Health, Heart, Lung and Blood Institute was completed most recently on 15 April 2022.

### 2.2. Study Measures and Outcomes

Dietary data were obtained using the CARDIA Diet History Questionnaire administered by the trained CARDIA staff and we only considered the dietary data from examination cycle 7 (year 20 of follow-op) when the cohort was between the ages of 38 and 50. The CARDIA staff was trained to conduct the diet interviews such that the participants were asked open-ended questions, which interrogated the intake of 100 food categories that detailed 1609 specific food items potentially consumed over the previous month [22]. Averaging of the daily intake of fructose and sodium was completed by the CARDIA personnel on assumption that each month had 30.42 days. Specific nutrient amounts were calculated with reference to the Food Table (Version 20) from the Nutrition Coordinating Center at the University of Minnesota. The Nutrition Working Group was appointed by the CARDIA investigators who oversaw all aspects of the Diet History method, including outliers and missing values. It was decided that all nutrient amounts and frequencies would be recorded as is, including the out-of-range values, and that missing values would not be imputed. Therefore, fructose and sodium amounts used in our study represent those recorded by the CARDIA personnel as indicated by the participants. Instead of using absolute numbers of grams of fructose per day, we converted this number into percent from daily calories by multiplying the number of grams of fructose by 4 (i.e., 4 calories per gram of fructose, to obtain calories from fructose), followed by division with the total number of daily calories consumed (to get the fraction of fructose per day) and multiplying by 100 (to get percent daily calories coming from fructose).

**Figure 1:**
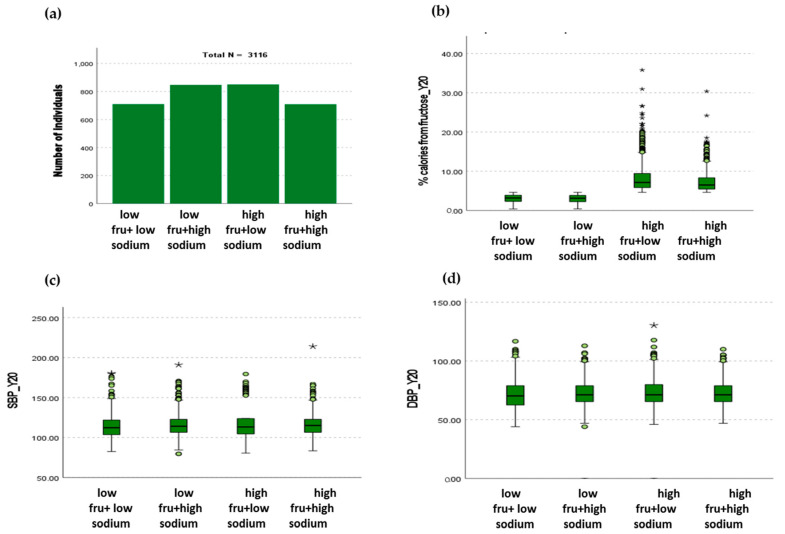
Relationship between fructose and sodium consumption and systolic and diastolic blood pressures. (**a**) The cohort was equally distributed across the four dietary groups according to fructose and sodium intake. (**b**) The average percentage of calories consumed in the low-fructose groups was 3.02 ± 1.01% and 3.00 ± 1.02% for low and high sodium, respectively. The average percentage of calories consumed in the high-fructose groups was 8.29 ± 3.76% and 7.34 ± 2.78% for low and high sodium, respectively. (**c**) The individuals in the low-fructose + high-sodium group had lower SBP compared to the individuals in low-fructose + high-sodium and high-fructose + high-sodium groups (*p* < 0.05). (**d**) The individuals in the high-fructose + low-sodium group had higher DBP compared to the low-fructose + low-sodium group (* *p* < 0.05).

Blood pressure measurement methods evolved throughout the examination cycles together with the available equipment. Thus, for examination cycle 1 (year 0, i.e., baseline) blood pressures were measured in triplicates with a random zero mercury sphygmomanometer by centrally trained personnel, while those for examination cycle 7 were completed with an Omron HEM907XL oscillometric monitor, by taking measurements on the right arm, in triplicates, following a 5 min rest period. When BPs are reported as absolute values (i.e., in Figure 1), the averages of the second and third values are represented. In order to mitigate potential inter-device differences, the trained CARDIA staff completed specific calibration procedures, as described before [23]. Here, we report the occurrence rate of blood pressure >130/80 mmHg, which is considered as clinical elevation as per the American Heart Association criteria [24]. At baseline on examination cycle 1, 13.1% of individuals (408 out of 3116) had BP >130/80 mmHg, independent of hypertension diagnosis, and on examination cycle 9 (year 30 of follow-up), this number rose to 14.9% (388 out of 3116). The hypertension diagnosis rate on examination cycle 1 was 13.1% and it increased to 37.9% 30 years later, on examination cycle 9. 

Echocardiography was completed on examination cycle 9 using a standardized procedure across all centers. Full echocardiograms were obtained with a 2-D M-Mode and Doppler ultrasonography. The reading of the echocardiograms was centralized and completed by expertly trained ultrasonographers. Peak mitral inflow diastolic velocities were measured as described before [25]. Coronary artery calcification was measured with cardiac multidetector computed tomography [26] and the corresponding Agatston scores were obtained [27]. Urinary albumin and creatinine were measured from an untimed spot urine sample centrally as described before [28]. 

Aerobic exercise activity was assessed via a validated, self-report questionnaire administered by the trained CARDIA staff [29]. The participants were asked if they consistently engaged in at least 60 min per month, over the previous 12 months, in vigorous intensity exercise such as running, biking, swimming and racquet sports. 

We defined outcome variables of hypertensive target organ damage as diastolic dysfunction, coronary calcification and albuminuria. Target organ damage is a common complication of hypertension and has been shown to increase the risk of CVD, even in cases of controlled hypertension [30,31,32,33]. Diastolic dysfunction was defined as the ratio of early to late peak inflow mitral velocities (E/A ratio) greater than 1.2 or lower than 0.8 [34]. Coronary calcification was defined as Agatston score ≥100 [35]. Albuminuria was defined as urinary albumin: creatinine ratio (UACR) >30 mg/g [36].

### 2.3. Statistical Analysis

We used the Statistical Package for the Social Sciences (SPSS) version 29.01.0 to complete the statistical analyses. All continuous variables were assessed for skewness and kurtosis with the Kolmogorov–Smirnov test, and they largely displayed univariate normality. A total of 3116 individuals were included in the final analysis. The characteristics of the cohort are represented using numbers and percentages. All of the characteristics were derived from the CARDIA visit measurements or sourced from CARDIA staff-administered questionnaires, while race and sex were self-reported. We used the cut-off value of significance for *p*-values < 0.05. We divided the cohort into four groups according to the amount of dietary fructose and sodium they were consuming at examination cycle 7 (year 20 of follow-up). For fructose groupings, an individual was placed in the *high-fructose group* if consuming the amount of fructose in the upper 50th percentile of the whole cohort. Otherwise, if an individual was consuming the amount of fructose corresponding to the lower 50th percentile, they were placed in the *low-fructose group*. Similarly, for sodium groupings, individuals were placed in the *high-sodium group* if consuming the amount of sodium corresponding to the upper 50th percentile of the cohort, or the *low-sodium group* if consuming the amount of sodium corresponding to the lower 50th percentile. The four possible permutations of the fructose and sodium variables gave rise to four groups: low fructose + low sodium (*n* = 710), low fructose + high sodium (*n* = 847), high fructose + low sodium (*n* = 850) and high fructose + high sodium (*n* = 709). Differences in systolic blood pressure (SBP) and diastolic blood pressure (DBP) at examination cycle 7 (year 20 of follow-up) across the four dietary groups were evaluated with the Kruskal–Wallis test. All other variables from examination cycles 0, 7 (follow-up year 20) and 9 (follow-up year 30) and all-cause mortality (follow-up year 35) were assessed for differences across the four dietary groups using the Pearson χ^2^ test. For the main analysis, we used binary logistic regression and the corresponding odds ratios for factors associated with outcomes of target organ damage: diastolic dysfunction (abnormal E/A [34]), coronary calcification (Agatston score ≥ 100 [35]) and albuminuria (UACR > 30 mg/g [36]). 

## 3. Results

### 3.1. Dietary Fructose and Sodium Associations with Systolic (SBP) and Diastolic Blood Pressure (DBP)

A total of 3116 CARDIA participants were included in this study. They were divided into four groups according to the amount of dietary fructose and sodium they were consuming at examination cycle 7 (follow-up year 20), as described in detail in Section 2. The four groups were low fructose + low sodium (*n* = 710), low fructose + high sodium (*n* = 847), high fructose + low sodium (*n* = 850) and high fructose + high sodium (*n* = 709) (Figure 1a). The amount of fructose expressed as percent total daily calories in the low-fructose group was 3.02 ± 1.01% in combination with low sodium (2320 ± 510 mg) and 3.00 ± 1.02% in combination with high sodium (4809 ± 2120 mg) and the amount of fructose in the high-fructose group was 8.29 ± 3.76 in combination with low sodium (2139 ± 599 mg) and 7.34 ± 2.78% in combination with high sodium (4730 ± 2005 mg) (Figure 1b). Individuals in the low-fructose + low-sodium group were found to have lower SBP compared to those in low-fructose + high-sodium and high-fructose + high-sodium groups (*p* < 0.05) (Figure 1c). DBP was found to be higher in individuals in the high-fructose + low-sodium group compared to those in the low-fructose + low-sodium group (*p* < 0.05) (Figure 1d). 

### 3.2. Characteristics of the Cohort Stratified according to Fructose and Sodium Consumption

We studied 1343 males and 1773 females from the CARDIA cohort, whose characteristics at examination cycle 1 (baseline) and examination cycles 7 and 9 are presented in Table 1. Dietary patterns were different between males and females as well as between individuals of Black and White race. There were more females in the low sodium groups, irrespective of fructose (*p* < 0.001). There were more individuals of White race compared to those of Black race in the low-fructose groups, irrespective of sodium. Although age and BMI were statistically significant among the four dietary groups, these differences are effectively insignificant (i.e., difference in 0.5 BMI points and 0.9 years). The proportion of individuals with blood pressure over 130/80 mmHg, which is defined as stage I hypertension by the current AHA guidelines, at baseline when the median age of the cohort was 26 years, was higher in the high-sodium groups both with low and high fructose (14.8 and 14.5%), and it was the lowest in the low-fructose + low-sodium group (10.4%, *p* = 0.044). 

The dietary data used in this study were obtained from the examination cycle 7, when the cohort’s median age was 46 years. The amounts of fructose that the individuals were consuming in the low-fructose group are presented in the graphical form in Figure 1, and the exact numbers are shown in Table 1. The average percentage of calories coming from fructose was 3.0 ± 1.0% for both the low- and high-sodium groups. The average percentage of calories coming from fructose in the high-fructose group in combination with low sodium was 8.3 ± 3.8% and 7.3 ± 2.8% in combination with high sodium. Individuals who consumed high sodium also consumed more potassium compared to the low-sodium groups (*p* < 0.001). HbA1c was similar across all groups at this time point. 

The outcome variables used in this study were obtained from examination cycle 9, at which time the cohort’s median age was 56 years. SBP was statistically significantly different across the groups (*p* = 0.004), with the low-fructose + low-sodium group having the lowest SBP (117 ± 15 mmHg) and the low-fructose + high-sodium and high-fructose + low-sodium groups having the highest SBP (120 ± 15 and 120 ± 17 mmHg, respectively). BMI was also different across the groups (*p* = 0.001) with the highest in the high-fructose + low-sodium group (31.0 ± 7.1) and the lowest in the low-fructose + low-sodium group (29.4 ± 6.2). The highest hypertension diagnosis rate occurred in the high-fructose + low-sodium group (41.2%) and differed significantly across the groups (*p* = 0.012). UACR > 30 g/mg and abnormal E/A were the highest in the high-fructose + high-sodium groups (9.8% and 18.2%, respectively, and were different across the groups (*p* = 0.016 and 0.042, respectively), while Agatston score was the highest in the high-sodium groups, irrespective of fructose (12. 7% and 8.7% in low and high fructose, respectively, *p* < 0.001). The incidence of CVD, ESRD and all-cause mortality did not differ across the groups.

### 3.3. Target Organ Damage According to Fructose and Sodium Consumption

Our outcome variables of target organ damage were defined as abnormal E/A for diastolic dysfunction [34], Agatston score ≥ 100 [35] for coronary calcification and UACR > 30 g/mg for albuminuria [36]. We completed logistic regression analyses for factors associated with the outcome variables by creating two models. Model 1 was adjusted for age, sex, race and the diagnoses of hypertension, diabetes, heart disease, high cholesterol and kidney disease (Table 2). The goal of this analysis was to highlight demographic and disease predictor differences among the four dietary groups according to fructose and sodium consumption. In Model 2, we intended to investigate whether the addition of aerobic physical activity in the form of running, biking, swimming and/or racket sports that the participants engaged in during mid-life altered the associations of demographic and disease predictors of target organ damage (Table 3). 

#### 3.3.1. Diastolic Dysfunction—Abnormal E/A

Participants in the low-fructose + low-sodium group were found to be protected from diastolic dysfunction if they belonged to the group of White race (OR = 0.497, *p* < 0.001, Table 2). This protection was lost, however, with increasing sodium and/or fructose. Model 2 showed the same decreased risk of diastolic dysfunction in individuals of White race eating low fructose and low sodium (OR = 0.510, *p* = 0.016, Table 3), while it also uncovered another protective effect of female sex in the high-fructose + high-sodium group (OR = 0.586, *p* = 0.037, Table 3).

#### 3.3.2. Coronary Calcification—Agatston Score ≥100

In Model 1 (Table 2), female sex was found to be protective across all four fructose and sodium groups from coronary calcification (low-fructose + low-sodium group: OR = 0.149, *p* < 0.001; low-fructose + high-sodium group: OR = 0.207, *p* < 0.001; high-fructose + high-sodium group: OR = 0.381, *p* = 0.004; high-fructose + high-sodium group: OR = 0.356, *p* = 0.008). Hypertension diagnosis in young adulthood was significantly associated with coronary calcification in the low-fructose + low-sodium group (OR = 6.520, *p* < 0.001), but this association was lost in the other dietary groups. Upon the addition of physical activity as a covariate in Model 2 (Table 3), no changes were observed in terms of risks of coronary calcification. Female sex was still protective across all four dietary groups (low-fructose + low-sodium group: OR = 0.129, *p* < 0.001; low-fructose + high-sodium group: OR = 0.201, *p* < 0.001; high-fructose + high-sodium group: OR = 0.343, *p* = 0.002; high-fructose + high-sodium group: OR = 0.322, *p* = 0.005. Hypertension diagnosis in young adulthood was significantly associated with coronary calcification in the low-fructose + low-sodium group only (OR = 7.034, *p* < 0.001).

#### 3.3.3. Albuminuria—UACR >30 mg/g

Individuals of White race were found to be protected from albuminuria if they belonged to the group with high sodium, irrespective of fructose (low fructose + high sodium: OR = 0.307, *p* < 0.001 and high fructose + high sodium: OR = 0.331, *p* = 0.001). Additionally, female sex was also associated with a lower risk of albuminuria in individuals belonging to the low-fructose groups only (low fructose + low sodium: OR = 0.401, *p* = 0.017 and low fructose + high sodium: OR = 0.475, *p* = 0.013). Hypertension diagnosis in participants’ young adulthood (i.e., in their mid-twenties) increases the risk of albuminuria 3-fold by the time the participants were in their mid-life (i.e., mid-forties), with OR = 3.238, *p* = 0.006, only if they were in the high-fructose + high-sodium group. Once we adjusted the model for physical activity (Table 3), the risk was still lower in the participants of White race in both high-sodium groups (OR = 0.345, *p* < 0.001 for low fructose + high sodium and OR = 0.327, *p* < 0.002 for high fructose + high sodium). Physical exercise also appears to be protective for albuminuria in females in all diet groups but the high fructose + high sodium, where this protection was lost. The risk of hypertensive filtration barrier damage was still found to be over 3-fold higher in individuals in the high-fructose + high-sodium group and no other permutation of fructose and sodium (OR = 3.308, *p* = 0.007). 

## 4. Discussion

This retrospective observational study was designed to investigate the relationship between different combinations of the upper and lower 50th percentile of dietary fructose and sodium with blood pressure and target organ damage in middle-aged Black and White adults in the CARDIA cohort. We found that those individuals consuming sodium in the upper 50th percentile of the cohort in combination with both lower and upper 50th percentiles of fructose had higher SBP compared to the individuals consuming both fructose and sodium in the lower 50th percentile (Figure 1c). We also found that the amounts of dietary fructose and sodium that the participants consumed when they were in their forties posed a different risk for hypertensive target organ damage 10 years later. We found no impact on diastolic dysfunction across the four dietary groups in our cohort. Coronary calcification risk was increased in the low-fructose and low-sodium group, whereas female sex was protective across all four groups. Risk of albuminuria was increased 3-fold in the high-fructose + high-sodium group only. Additionally, female sex was associated with decreased risk of albuminuria only in the low-fructose groups, and White race was protective only in the high-sodium groups.

To our knowledge, this study is the first to investigate how the different combinations of fructose and sodium are associated with the risk of cardiovascular and renal diseases in humans. Data from the rodent studies informed on how feeding 20% fructose and 4% NaCl chow for three weeks resulted in increases in mean arterial pressure and renal sympathetic nerve activity, which was not observed in rats fed 20% glucose in combination with 4% NaCl [6]. Likewise, the same moderate amount of fructose and sodium feeding for four weeks in rats has shown to result in an increase in aortic stiffness, diastolic dysfunction and reduced vascular distensibility [8], and that at least part of the mechanism involves an increase in renal sympathetic nerve activity. Additionally, young rats (equivalent of human adolescence) fed a 20% fructose + 4% NaCl diet were found to be susceptible to salt sensitivity of blood pressure and a decreased glomerular filtration rate when high sodium was re-introduced to them later in life (equivalent of human mid-life) [9,10], suggesting lasting consequences on salt sensitivity for both the cardiovascular and renal systems caused by dietary fructose. Importantly, all these rodent studies were conducted using the moderately increased dietary fructose (i.e., 20%) over short period of time (i.e., 3–4 weeks), rather than massively high fructose that is not representative of human consumption (i.e., 60% of daily calories) with exposure times of 6 months and longer. Recently, published data from the Jackson Heart study showed that Black individuals who regularly consumed high-fructose corn syrup-sweetened beverages had two times the risk of coronary heart disease (CHD) compared to never/seldom consumers and that those who consumed more than three such drinks per day had 2.5–3 times the risk of CHD [37]. This study, however, only evaluated the risk of CHD according to fructose-sweetened beverage consumption, whereas we evaluated the impact of all fructose from all dietary sources, expressed as percent of total daily calories. We calculated the amount of fructose in the high-fructose groups to be about 8% of the total daily calories (Figure 1b), and for a standard 2000 calorie per day diet, this equates to ~40 g of fructose, the amount found in two cans of soda. Therefore, when interpreting our data, and to provide a more direct comparison with the Jackson Heart data, one can visualize our results as comparisons between those ingesting less than two cans of soda (low-fructose group) and those ingesting more than two cans of soda per day (high-fructose group). Notwithstanding, both our previously published work in the CARDIA cohort showing an increased risk of CVD incidence with all dietary fructose consumption in Black and White individuals [12] and the current investigation are in agreement with the data from the Jackson Heart study [37] in that increasing amounts of dietary fructose are associated with increasing risk of cardiovascular disease and also renal disease, as we can now highlight with the current study.

Distributions of race and sex differed across the four dietary groups. A higher proportion of Black individuals compared to White individuals were in the high-fructose groups, whereas a higher percentage of males compared to females were in the high-sodium groups (*p* < 0.001 for both). Such trends were also observed on the national level, and according to the National Health Interview Survey, the intake of added sugars was highest among Black Americans, in both male and female categories [38]. Likewise, sodium intake has been shown to be higher in men compared to women in the recently published cross-sectional analysis of the 2011–2016 National Health and Nutrition Examination Survey (NHANES) [39]. It has also been suggested that sodium per se may not be the best predictor of health outcomes, but rather a sodium to potassium ratio (Na:K), and according to the World Health Organization and multiple published studies, an Na:K ≤1 is considered ideal for cardiovascular health and overall health [40,41,42,43,44]. Indeed, we also previously published in the middle-aged men of the CARDIA who consumed more sodium than women, that intake of potassium was inversely proportional to the vascular aging index, which we calculated using the carotid intima media thickness and carotid pulse wave velocities [12]. Interestingly, in the present investigation, we found that in both low sodium + low fructose and low sodium + high fructose, the Na:K was ≤1 (0.99 and 0.88, respectively), while Na:K was >1 in high-sodium + low-fructose (1.25) and high-sodium + high-fructose (1.11) groups, and it is in the latter two that we found an increased risk of hypertensive target organ damage in the kidney with Black race being a significant covariate. Furthermore, in the present study, the impetus for adding fructose as a covariate to sodium was to uncover another layer of the dietary puzzle that is associated with cardiovascular and renal outcomes amongst middle-aged Americans, since age contributes to increasing morbidity and mortality risk, because typically people do not consume high sodium in isolation, but rather in combination with either high fat or high fructose, or both, and in the present investigation we focus on fructose combinations. Perhaps one of the most demonstrative pieces of evidence that sodium is not necessarily the only culprit comes from the SODIUM-HF trial, an international trial with chronic heart failure patients, and arguably the most challenged to achieve natriuresis, who were either placed on a low-sodium diet (median intake of 1658 mg/day) or standard of care (median intake 2073 mg/day) in which no differences were observed in cardiovascular-related admissions to hospital, cardiovascular-related emergency department visits or all-cause death within 12 months [45]. It is nevertheless recognized that both groups in the SODIUM-HF trial were consuming <2300 mg/day, which is much lower than the average CARDIA participant (3496 mg/day) who were importantly also devoid of heart failure.

Target organ damage incidence investigated in this study included diastolic dysfunction, coronary calcification and albuminuria, and all three differed across the fructose and sodium groups. The high-fructose + high-sodium group had the highest prevalence of abnormal E/A (18.2%, *p* = 0.042) and UACR > 30 mg/g (9.8%, *p* = 0.016). The Agatston score ≥100 was higher in the high-sodium groups (12.7% and 8.7%, respectively, *p* < 0.001) compared to the low-sodium groups. Using a model adjusted for age, high cholesterol, heart disease, diabetes and kidney disease diagnoses, we observed some effects of race and sex on the risks examined in these organs. For example, individuals of White race were found to be at a lower risk of diastolic dysfunction only when they were consuming low fructose and low sodium (OR = 0.497, *p* = 0.001), and as soon as they increased either fructose, or sodium, or both, this protection disappeared. Likewise, individuals of White race were found to be at a lower risk of UACR >30 mg/g only if they belonged to the low-sodium groups, indicating that racial disparities for cardiovascular and renal disease susceptibility no longer exist when individuals increase sodium and fructose consumption. Females were also found to be protected from coronary calcification with decreased risk across all combinations of fructose and sodium (Table 2). A lower incidence of coronary artery calcification was also reported among women in a middle-aged Dutch cohort [46]. This may be explained by the differences in coronary plaque composition, where women were found to have more lipid-rich, less-calcified plaques compared to men [47,48]. Similarly, protection against albuminuria seemed to have been afforded to individuals of the female sex only if they were consuming low fructose, because these protections disappeared in the high-fructose groups, independent of sodium intake, suggesting a significant impact of higher dietary fructose intake on risk of albuminuria a decade later. Hypertension age onset has been reported to play a major role in hypertensive target organ damage in the CARDIA cohort, with the age of onset <35 years being associated with the highest risk of left ventricular hypertrophy, coronary artery calcification and albuminuria assessed on the CARDIA examination cycle 8 (when the cohort was just approaching or entering their fifties) [49]. Here, we further hone in on the risk stratified according to the dietary patterns for fructose and sodium. We also found that hypertension onset at the age < 35 years more than triples (OR = 3.328, *p* = 0.006) the risk of albuminuria (which we measured at examination cycle 9, when the cohort was in their mid-fifties) but only in individuals who were eating high fructose and high sodium 10 years prior to that (Table 2). We also evaluated the risk of target organ damage using a model further adjusted for exercise, including running, biking, swimming and racket sports (Table 3) since it was recently published that cardiorespiratory fitness is inversely proportional to all-cause mortality in a population of U.S. veterans aged 30 to 95 years [50]. In this study, the least fit individuals were at a four-fold increased risk of mortality; however, there was no consideration of the participants consumption of fructose, sodium or any other dietary aspect. After we adjusted the model for the physical activity types that have been shown to result in increased cardiorespiratory fitness [51], most of the target organ damage risks remained unchanged (Table 3), suggesting that one cannot out-exercise poor dietary choices, a finding that was corroborated by our previously published work in the CARDIA cohort regarding vascular aging and CVD incidence [12]. One change that did appear in the model adjusted for exercise was that female sex was associated with about a 50% reduced risk of diastolic dysfunction in the high-fructose + high-sodium group (0.586, *p* = 0.037) as well as in the high-fructose + low-sodium group for albuminuria (OR = 0.498, *p* = 0.050), indicating that aerobic exercise in middle-aged women may afford further protection against cardiovascular and renal morbidity associated with dietary fructose and sodium.

## 5. Conclusions

In this retrospective cohort study, we found that sodium alone may not be the only culprit for blood pressure regulation and hypertensive target organ damage, but rather other dietary components, such as fructose, may increase the susceptibility to salt sensitivity of blood pressure and consequent target organ damage. Individuals in the low-fructose + low-sodium group were found to have a lower SBP compared to those in the low-fructose + high-sodium and high-fructose + high-sodium groups. DBP was found to be higher in individuals in the high-fructose + low-sodium group compared to those in the low-fructose + low-sodium group. In the present investigation, we explored the impact of sodium and fructose intake consumed when the participants were in their forties on target organ damage one decade later, when the participants were in their fifties. Race was found to be a significant covariate in determining the risk of diastolic dysfunction, where participants in the low-fructose + low-sodium group were found to be protected from diastolic dysfunction if they belonged to the group of White race. This protection was lost, however, with increasing sodium and/or fructose, suggesting that protections/susceptibilities inherent to the demographic/genetic characteristics are importantly influenced by the diet. Likewise, individuals of White race were found to be protected from albuminuria if they belonged to the groups with high sodium, irrespective of fructose. Additionally, female sex was also associated with a lower risk of albuminuria in individuals belonging to the low-fructose groups only. Finally, the highest risk for albuminuria was found for individuals belonging to the high-fructose + high-sodium group who were diagnosed with hypertension in their twenties.

## Figures and Tables

**Table 1:** Characteristics of the cohort.

		Total Cohort	Low FRU + Low SOD	Low FRU + High SOD	High FRU + Low SOD	High FRU + High SOD	*p*-Value
**Examination cycle 1: baseline**							
Race	Black	1455 (46.7)	245 (34.5)	297 (35.1)	536 (63.1)	377 (53.2)	<0.001
	White	1661 (53.3)	465 (65.5)	550 (64.9)	314 (36.9)	332 (46.8)	
Sex	Male	1343 (43.1)	213 (30.0)	484 (57.1)	235 (27.6)	411 (58.0)	<0.001
	Female	1773 (56.9)	497 (70.0)	363 (42.9)	615 (72.4)	298 (42.0)	
Age		25.1 (3.6)	25.4 (3.5)	25.0 (3.5)	25.2 (3.7)	24.6 (3.6)	<0.001
BMI		24.3 (4.7)	24.1 (4.8)	24.5 (4.3)	24.6 (5.1)	24.1 (4.5)	0.006
HBP Dx		276 (8.9)	62 (8.7)	84 (9.9)	72 (8.5)	58 (8.2)	0.653
High Cholesterol		62 (2.0)	16 (2.3)	16 (1.9)	15 (1.8)	62 (2.0)	0.895
Heart Disease		197 (6.4)	44 (6.3)	46 (5.5)	59 (7.0)	48 (6.8)	0.562
Diabetes		22 (0.7)	6 (0.8)	7 (0.8)	6 (0.7)	3 (0.4)	0.767
Kidney Disease		122 (3.9)	30 (4.3)	31 (3.7)	35 (4.1)	26 (3.7)	0.908
BP > 130/80 mmHg		408 (13.1)	74 (10.4)	125 (14.8)	106 (12.5)	145 (14.5)	0.044
**Examination cycle 7: year 20 of follow-up**							
Smoking		592 (19.0)	117 (16.5)	177 (20.9)	133 (15.6)	165 (23.3)	<0.001
BMI		29.17 (6.8)	28.48 (6.5)	29.10 (6.5)	29.93 (7.2)	29.02 (6.7)	<0.001
SBP		115 (15)	113 (15)	116 (14)	115 (16)	116 (14)	<0.001
DBP		72 (11)	71 (11)	72 (11)	73 (12)	72 (11)	0.027
Fructose (% calories)		5.4 (3.5)	3.0 (1.0)	3.0 (1.0)	8.3 (3.8)	7.3 (2.8)	<0.001
Sodium (mg)		3496 (1981)	2320 (510)	4809 (2120)	2139 (599)	4730 (2005)	<0.001
Potasium (mg)		3210 (1611)	2321 (823)	3848 (1513)	2429 (1036)	4278 (1890)	<0.001
HbA1c		5.5 (0.9)	5.5 (0.9)	5.6 (1.0)	5.5 (0.8)	5.5 (0.8)	0.119
**Examination cycle 9 (year 30 of follow-up)**							
SBP		119 (18)	117 (15)	120 (15)	120 (17)	119 (15)	0.004
DBP		73 (11)	72 (11)	73 (10)	73 (11)	73 (11)	0.194
BMI		30.2 (6.7)	29.4 (6.2)	30.4 (6.5)	31.0 (7.1)	29.9 (6.5)	0.001
HbA1c		5.6 (0.6)	5.6 (0.5)	5.6 (0.5)	5.7 (0.65)	5.6 (0.4)	0.219
Runnung		627 (24.8)	142 (24.7)	175 (24.9)	152 (21.7)	158 (28.0)	0.168
Racquet sport		134 (5.3)	29 (5.0)	45 (6.4)	24 (3.4)	36 (6.4)	0.100
Biking		854 (33.6)	195 (33.9)	247 (35.2)	223 (31.9)	189 (33.5)	0.633
Swimming		423 (16.6)	98 (17.0)	123 (17.5)	91 (13.0)	111 (19.6)	0.028
HBP Dx		979 (37.9)	188 (32.5)	278 (39.0)	296 (41.2)	217 (38.0)	0.012
UACR > 30 mg/g		198 (7.7)	35 (6.0)	64 (9.0)	43 (6.0)	56 (9.8)	0.016
Agatston score ≥100		230 (9.1)	43 (7.5)	90 (12.7)	48 (7.0)	49 (8.7)	<0.001
E/A abnormal		391 (15.7)	79 (14.0)	92 (13.4)	120 (17.5)	100 (18.2)	0.042
BP > 130/80 mmHg		388 (14.9)	78 (13.3)	111 (15.5)	117 (16.2)	82 (14.2)	0.474
CVD		224 (7.2)	45 (6.3)	63 (7.4)	58 (8.2)	58 (8.2)	0.559
ESRD		24 (0.8)	7 (1.0)	5 (0.6)	6 (0.7)	6 (0.8)	0.828
All-cause mortality		168 (5.4)	39 (5.5)	43 (5.1)	51 (6.0)	35 (4.9)	0.781

FRU = fructose, SOD = sodium, HBP Dx = high blood pressure diagnosis, SBP = systolic blood pressure, DBP = diastolic blood pressure, UACR = urinary albumin creatinine ratio, E/A = ratio of early to late transmittal peak flow velocities, ESRD = end stage renal disease. Data are represented as number (percentages) for categorical variables and means (S.D.) for continuous variables.

**Table 2:** Target organ damage at examination cycle 9 (follow-up year 30), when the median age of the cohort was 56 years: Model 1.

	Low FRU + Low SOD	Low FRU + High SOD	High FRU + Low SOD	High FRU + High SOD
**E/A abnormal**				
White race	**0.497 [0.292–0.846], 0.001**	0.985 [0.607–1.598], 0.952	1.323 [0.860–2.034], 0.203	0.740 [0.462–1.185], 0.211
Female sex	1.623 [0.891–2.957], 0.114	1.356 [0.862–2.133], 0.188	0.949 [0.599–1.503], 0.822	0.644 [0.411–1.073], 0.095
HBP Dx bsl	0.949 [0.365–2.468], 0.915	0.687 [0.294–1.604], 0.386	0.687 [0.294–1.604], 0.386	1.777 [0.844–3.743], 0.130
**Agatston score ≥ 100**				
White race	1.318 [0.565–3.071], 0.523	0.755 [0.448–1.272], 0.291	1.270 [0.661–2.441], 0.474	1.327 [0.682–2.582], 0.405
Female sex	**0.149 [0.069–0.325], <0.001**	**0.207 [0.111–0.383], <0.001**	**0.381 [0.199–0.730], 0.004**	**0.356 [0.166–0.766], 0.008**
HBP Dx bsl	**6.520 [2.745–15.485], <0.001**	1.956 [0.948–4.038], 0.070	1.274 [0.412–3.938], 0.674	0.523 [0.119–2.293], 0.390
**UACR > 30 mg/g**				
White race	0.471 [0.216–1.027], 0.058	**0.307 [0.176–0.535], <0.001**	0.707 [0.352–1.463], 0.350	**0.331 [0.167–0.654], 0.001**
Female sex	**0.401 [0.189–0.848], 0.017**	**0.475 [0.265–0.853], 0.013**	0.532 [0.268–1.054], 0.070	0.804 [0.428–1.508], 0.496
HBP Dx bsl	1.734 [0.591–5.093], 0.317	1.749 [0.793–3.857], 0.166	2.273 [0.872–5.929], 0.093	**3.328 [1.390–7.544], 0.006**

Significant predictors are bolded for emphasis; FRU = fructose, SOD = sodium, HBP Dx bsl = high blood pressure diagnosis at baseline (median age 26 years), UACR = urinary albumin creatinine ratio, E/A = ratio of early to late transmittal peak inflow velocities.

**Table 3:** Target organ damage at examination cycle 9 (follow-up year 30), when the median age of the cohort was 56 years: Model 2.

	Low FRU + Low SOD	Low FRU + High SOD	High FRU + Low SOD	High FRU + High SOD
**E/A abnormal**				
White race	**0.510 [0.295–0.880], 0.016**	1.067 [0.649–1.754], 0.798	1.204 [0.764–1.896], 0.424	0.722 [0.443–1.174], 0.189
Female sex	1.691 [0.911–3.141], 0.096	1.341 [0.842–2.137], 0.216	0.940 [0.590–1.498], 0.795	**0.586 [0.355–0.967], 0.037**
HBP Dx bsl	0.942 [0.361–2.461], 0.903	1.151 [0.564–2.351], 0.699	0.701 [0.297–1.656], 0.418	1.585 [0.739–3.396], 0.237
**Agatston score ≥ 100**				
White race	1.443 [0.599–3.473], 0.414	0.822 [0.481–1.406], 0.475	1.266 [0.634–2.527], 0.503	1.516 [0.759–3.028], 0.238
Female sex	**0.129 [0.056–0.293], <0.001**	**0.201 [0.107–0.377], <0.001**	**0.343 [0.176–0.667], 0.002**	**0.322 [0.146–0.708], 0.005**
HBP Dx bsl	**7.034 [2.892–17.111], <0.001**	1.961 [0.944–4.071], 0.071	1.389 [0.446–4.328], 0.571	0.549 [0.123–2.460], 0.434
**UACR > 30 mg/g**				
White race	0.454 [0.200–1.033], 0.060	**0.345 [0.196–0.608], <0.001**	0.735 [0.341–1.585], 0.433	**0.327 [0.163–0.656], 0.002**
Female sex	**0.273 [0.122–0.641], 0.002**	**0.442 [0.242–0.806], 0.008**	**0.498 [0.249–0.999], 0.050**	0.794 [0.411–1.535], 0.493
HBP Dx bsl	1.797 [0.601–5.374], 0.294	1.731 [0.782–3.832], 0.176	2.032 [0.767–5.383], 0.154	**3.308 [1.394–7.851], 0.007**

Significant predictors are bolded for emphasis; FRU = fructose, SOD = sodium, HBP Dx bsl = high blood pressure diagnosis at baseline (median age 26 years), UACR = urinary albumin creatinine ratio, E/A = ratio of early to late transmittal peak inflow velocities.

## Data Availability

The data presented in this study are available on request from the corresponding author. The data are not publicly available due to necessitation of Research Materials Distribution Agreement with the National Institutes of Health, Heart Lung and Blood Institute.
